# Hepatic Effects of Pharmacological Doses of Hydroxy-Cobalamin[c-lactam] in Mice

**DOI:** 10.1371/journal.pone.0171026

**Published:** 2017-01-30

**Authors:** Patrizia Haegler, David Grünig, Benjamin Berger, Luigi Terracciano, Stephan Krähenbühl, Jamal Bouitbir

**Affiliations:** 1 Division of Clinical Pharmacology & Toxicology, University Hospital, Basel, Switzerland; 2 Department of Biomedicine, University of Basel, Basel, Switzerland; 3 Swiss Center of Applied Human Toxicology, SCAHT, Basel, Switzerland; 4 Department of Molecular Pathology, Institute for Pathology, University Hospital, Basel, Switzerland; East Tennessee State University, UNITED STATES

## Abstract

The vitamin B_12_ analog hydroxy-cobalamin[c-lactam] (HCCL) impairs hepatic mitochondrial protein synthesis and function of the electron transport chain in rats. We aimed to establish an *in vivo* model for mitochondrial dysfunction in mice, which could be used to investigate hepatotoxicity of mitochondrial toxicants. In a first step, we performed a dose-finding study in mice treated with HCCL 0.4 mg/kg and 4 mg/kg i.p. for two to four weeks. The plasma methylmalonate concentration was strongly increased at 4 mg/kg starting at three weeks of treatment. We subsequently treated mice daily with 4 mg/kg HCCL i.p. for three weeks and characterized liver function and histology as well as liver mitochondrial function. We found an increase in liver weight in HCCL-treated mice, which was paralleled by hepatocellular accumulation of triglycerides. In liver homogenate of HCCL-treated mice, the complex I activity of the electron transport chain was reduced, most likely explaining hepatocellular triglyceride accumulation. The activity of CPT1 was not affected by methylmalonyl-CoA in isolated liver mitochondria. Despite impaired complex I activity, mitochondrial superoxide anion production was not increased and the hepatocellular glutathione (GSH) pool was maintained. Finally, the mitochondrial DNA content was not altered with HCCL treatment. In conclusion, treatment of mice with HCCL is associated with increased liver weight explained by hepatocellular triglyceride accumulation. Hepatocellular fat accumulation is most likely a consequence of impaired activity of the mitochondrial electron transport chain. The impairment of complex I activity is not strong enough to result in ROS accumulation and reduction of the GSH stores.

## Introduction

Methylmalonyl-CoA (MM-CoA) mutase catalyzes the conversion of MM-CoA to succinyl-CoA, which is an essential step in propionic acid metabolism [1–3]. MM-CoA mutase is mainly expressed in liver, kidney and heart, but also in the central nervous system [4, 5]. The gene coding for MM-CoA mutase (*MUT* gene, MIM *609058) is located on chromosome 6 [6] and encodes a protein containing 750 amino acids. After biosynthesis in the endoplasmic reticulum, MM-CoA mutase has to be transported into the mitochondrial matrix [7], where propionyl-CoA and MM-CoA are produced mainly by the breakdown of odd-chain fatty acids, branched-chain amino acids and of cholesterol. A decreased or lacking activity of MM-CoA mutase results in methylmalonic aciduria, an inborn error of metabolism, which mainly affects newborns and children [1–3].

MM-CoA mutase requires adenosylcobalamin (AdoCbl) as a prosthetic group, which is formed by the enzyme ATP:Cob(I)alamin adenosyltransferase [8]. The Co-carbon bond between the cobalt atom of vitamin B12 and the adenosyl moiety is weak, allowing homolytic cleavage with the formation of a deoxyadenosyl radical, which is essential for the rearrangement of MM-CoA to succinyl-CoA [9]. In accordance with the essential role of vitamin B12 in this reaction, vitamin B12 deficiency is associated with decreased activity of MM-CoA mutase, leading to increased concentrations of methylmalonate and of other propionyl-CoA-derived metabolites such as 2-methylcitrate in plasma and urine [10, 11].

Animal models for methylmalonic acidura include knock-out mice [12], transgenic mice hemizygous for the human gene on a homozygous knockout background [13] and rats treated with the vitamin B12 analog hydroxyl-cobalamin[c-lactam] (HCCL) [14, 15]. Rats treated for several weeks with continuous subcutaneous infusion of HCCL developed methylmalonic aciduria [16] as well as proliferation [15] and impaired function of complexes III and IV of liver mitochondria [14]. Further studies suggested that impaired processing of mitochondrial RNA, leading to a deficiency of mitochondrial mRNA, was the principle reason for HCCL-associated hepatic mitochondrial dysfunction [17].

So far, the effect of HCCL on liver mitochondria has been investigated *in vivo* only in rats, but not in mice. We were interested to establish this model of hepatic mitochondrial dysfunction also in mice for several reasons. First, HCCL is expensive and the dose in mice could be expected to be lower than in rats. Second, one of our goals was to create a simple, reproducible model of a hepatic mitochondrial disease in mice. We planned to use this model for toxicological studies of established or suspected mitochondrial toxicants, since underlying mitochondrial diseases can aggravate cellular and/or organ toxicity of mitochondrial toxicants [18–20]. Furthermore, we also wanted to study the consequences of the expected impairment of the mitochondrial respiratory chain on hepatic function and morphology in more detail.

In order keep the model as simple as possible, we decided to administer HCCL by repetitive intraperitoneal injections and not by insertion of osmotic minipumps as performed in the previous studies with rats [14, 15] and mice [21]. We first conducted a dose finding study in order to determine the dose leading to a significant elevation of plasma MMA in mice treated with repetitive intraperitoneal injections of HCCL. After that, we treated mice with the established treatment regimen and investigated liver morphology and function as well as the function of the respiratory chain and of other aspects of liver mitochondria of these animals.

## Materials and Methods

### Chemicals

HCCL was synthesized on request from ReseaChem (Burgdorf, Switzerland) as described by Stabler et al. [22]. The manufacturer declared a 99% purity of HCCL by high-performance liquid chromatography (HPLC) and confirmed the structure by ^1^H-NMR analysis. Stock solutions were prepared in water and stored at -20°C. All other chemicals used were purchased from Sigma-Aldrich or Fluka (Buchs, Switzerland), except where indicated.

### Animals

The experiments described were performed according to the guide for the care and use of laboratory animals at Basel University and were approved by the Animal Care and Use Committee of Basel-Stadt in Switzerland (Cantonal Veterinary Office: License 2623). Male C57BL/6 mice were purchased from Charles River Laboratories (Sutzfeld, Germany) and were adult age-matched (7-weeks old). They housed in a standard facility with 12h light-dark cycles and controlled temperature (21–22°C). The mice were fed with a standard pellet chow and water *ad libitum*. After the treatment, all mice from this study were anaesthetized with an intraperitoneal application of ketamine (100 mg/kg) and xylazine (10 mg/kg) and then blood was collected into heparin-coated tubes by an intracardiac puncture. Finally all mice were sacrificed by heart and liver excision, followed by exsanguination.

### Dose finding study

After one week of acclimatization, 36 animals were randomly assigned to one of the treatment groups. The mice were treated daily intraperitoneally (i.p.) for 2 weeks (n = 12), 3 weeks (n = 12) or 4 weeks (n = 12) with two different doses of HCCL (0.4 mg/kg/d and 4 mg/kg/d) or saline. The HCCL doses were chosen according to previous studies in rats using a dose of 48 μg/rat per day [14, 15].

### Determination of methylmalonic acid

The determination of methylmalonic acid was accomplished by the LC-MS/MS analysis using the ClinMass^®^ Complete Kit (Recipe Chemicals + Instruments GmbH, Munich, Germany). Chromatographic separation was performed using a Shimadzu HPLC (Shimadzu AG, Reinach, Switzerland). The LC system was interfaced with a triple quadruple mass spectrometer (API4000, AB Sciex, Concord, Canada) equipped with an electrospray ionization (ESI) source. Methylmalonic acid and methylmalonic acid-d3 were detected by selected reaction monitoring (SRM) with a transition of 116.9 → 72.8/54.9 m/z and 119.9 → 76.0 m/z, respectively. Inter-assay accuracy (determined as the percentage bias) for control samples ranged from -4.1 to 7.2%, and inter-assay precision (determined as the relative standard deviation) was lower than 7.9%. The lower limit of quantification (LLOQ) was 2.48 ng/mL for methylmalonic acid.

### HCCL treatment

After one week of acclimatization, the mice were divided into two groups: 1) 10 mice (saline group) treated daily with the same amount of saline and served as a control and 2) 12 mice (HCCL group) treated with HCCL by intraperitoneal injection at 4 mg/kg body weight/day for 3 weeks. Animals and food intakes were weighed every two days.

### Sample collection

Plasma was separated by centrifugation at 3,000g for 15 min. One part of the liver was collected and conserved in ice-cold MiR06 (mitochondrial respiration medium containing 0.5 mM EGTA, 3 mM MgCl_2_, 60 mM K-lactobionate, 20 mM taurine, 10 mM KH_2_PO_4_, 20 mM HEPES, 110 mM sucrose, 1 g/L fatty acid-free bovine serum albumin [BSA] and 280 U/ml catalase, pH 7.1) before being transferred to the pre-calibrated oxygraph chambers.

The other part of the liver was frozen in liquid nitrogen immediately after excision. Since the mice were anesthetized, tissues were obtained from living animals, and the time between sampling and freezing was only a few seconds. Samples were kept at −80°C until analysis.

### Plasma parameters

Aspartate aminotransferase (AST) and total bilirubin were analysed using a commercially available colorimetric assay kit (Abcam, Cambridge, UK).

### Histological analysis of liver tissue

Cross sections (10 μm) with a cryostat microtome were fixed with paraformaldehyde 4%. All sections were stained with hematoxylin eosin for a coarse assessment of tissue damage.

The glycogen content was investigated using periodic acid Schiff staining on paraformaldehyde fixed sections. For that, sections were incubated in 0.5% periodic acid for 8 min, washed in running water for 5 min and in distilled water twice for several seconds. Afterwards, they were incubated in Schiff ‘s solution for 10 min, followed by 0.5% sodium metabisulfite 3 times for 3 min each. Finally, cover slips were placed on glass slides with a mounting medium and observed under a light microscope.

Lipid accumulation was investigated by Oil red O staining of frozen liver sections. Oil red O was freshly diluted (3:2 in distilled water) from a stock solution in isopropanol (0.5 g in 100 ml) and sections were incubated for 15 min. After incubation, the slides were rinsed with 60% isopropanol, counterstained with haematoxylin and coverslipped in aqueous mountant.

The stained sections were examined by light microscopy and investigated for pathological changes in the liver with the examiner blinded regarding the treatment of the animals. Graduation of the changes was performed using a semi-quantitative histological score ranging from 0 (no glycogen of fat) to 4 (highest degree of glycogen or fat accumulation).

### Ki-67 protein content

Liver Ki-67 protein content was measured by using a commercially available mouse antigen Ki-67 ELISA kit (Biomatik, Ontario, Canada) based on a sandwich ELISA. Results were calculated from a standard curve generated by a parametric logistic curve fit and expressed in nanograms per milligram liver. In brief, liver tissue (10 mg) was homogenized in 1 mL PBS with a Mikro-Dismembrator for 1 minute at 3000 rpm (Sartorius Stedim Biotech, Göttingen, Germany). Standard and samples (100 μL) were added to each well and incubated for 2 h at 37°C. Next, 100 μL of prepared detection reagent A was added and the samples incubated for 1 h at 37°C. After that, detection reagent B (100 μL) was added and the samples incubated for 30 min at 37°C. This was followed by the addition of substrate (90 μL) and incubation for 20 min at 37°C. Finally, 50 μL of stop solution was added. Absorbance at 450 nm was immediately analyzed in a Tecan M200 Pro Infinity plate reader (Männedorf, Switzerland).

### CPT1 activity assay

The activity of carnitine palmitoyltransferase (CPT1) 1a was assessed as the formation of palmitoyl-^14^C-carnitine from palmitoyl-CoA and ^14^C-carnitine as described by Kennedy et *al*. with some modifications [23]. 200 μg of frozen isolated mouse liver mitochondria were incubated for 10 min in 450 μL assay buffer (80 mM KCl, 50 mM MOPS, 10 mg/ml BSA defatted, 5 mM EGTA, 1.2 mM N-ethylmaleimide and water, pH 7.4) at 37°C on a thermomixer at 800 rpm together with treatments. The reaction was started by the addition of 50 μL radioactive substrate mix containing 20 mM L-carnitine, 1 μCi ^14^C-L-carnitine and 2 mM palmitoyl-CoA. The reaction was terminated after 10 min by adding 100 μL of 6% perchloric acid. Next, the samples were transferred into a 5 ml Eppendorf tube with 1.4 mL n-butanolsaturated distilled water and 800 μL n-butanol. After extraction, the upper (butanol) phase was transferred in a new tube containing 2 mL of butanol-saturated water. Finally, the upper butanol-phase containing the lipophilic palmitoyl-^14^C-L-carnitine was removed and the radioactivity was determined by liquid scintillation counting.

### Mitochondrial isolation and homogenate of liver

Fresh liver tissue was quickly removed and immersed in ice-cold isolation buffer (200 mM mannitol, 50 mM sucrose, 1 mM Na_4_EDTA, 20 mM HEPES, pH 7.4). Liver mitochondria were isolated by differential centrifugation as described previously [24]. The mitochondrial protein content was determined using the bicinchoninic acid protein assay reagent from Thermo Scientific (Wohlen, Switzerland).

Liver homogenate (250 mg fresh liver) was prepared with the PBI-Schredder HRR set (Oroboros instruments, Austria) according to the manufacturer’s instruction [25].

### Mitochondrial membrane potential

The mitochondrial membrane potential (Δψ_m_) was determined using the [phenyl-^3^H]-tetra-phenylphosphonium bromide uptake assay. Freshly isolated mouse-liver mitochondria (200 μg/mL) were washed with incubation buffer (137.3 mM sodium chloride, 4.74 mM potassium chloride, 2.56 mM calcium chloride, 1.18 mM potassium phosphate, 1.18 mM magnesium chloride, 10 mM HEPES, 1 g/L glucose, pH 7.4). Mitochondria were then incubated at 37°C in buffer containing 0.5 μL/ml [phenyl-^3^H]-tetra-phenylphosphonium bromide (40 Ci/mmol, Anawa trading SA, Wangen, Switzerland). After 15 min, the suspension was centrifuged and the mitochondrial pellet resuspended in fresh non-radioactive incubation buffer with test compounds inside. Non-radioactive tetra-phenylphosphonium was used as a positive control. Mitochondria were treated for 15 min at 37°C. Afterwards, mitochondria were centrifuged and resuspended in non-radioactive buffer. The radioactivity of the samples was measured on a Packard 1900 TR liquid scintillation analyzer.

### Enzyme complexes activity of the mitochondrial electron transport chain

The activity of the enzyme complexes was analyzed using an Oxygraph-2k high-resolution respirometer equipped with DatLab software (Oroboros Instruments, Innsbruck, Austria). Fresh liver homogenate (20 μL /chamber) was diluted with mitochondrial respiration medium MiR06 (mitochondrial respiration medium containing 0.5 mM EGTA, 3 mM MgCl_2_, 60 mM K-lactobionate, 20 mM taurine, 10 mM KH_2_PO_4_, 20 mM HEPES, 110 mM sucrose, 1 g/L fatty acid-free bovine serum albumin [BSA] and 280 U/mL catalase, pH 7.1) before being transferred to the pre-calibrated oxygraph chambers.

In a first run, complexes I and III were analyzed using L-glutamate/malate (10 and 5 mM respectively) as substrates followed by the addition of ADP (2 mM). Then, we added oligomycin (2.5 μM) for the determination of the leak-state respiration. This was followed by the addition of FCCP (1 μM) to reach a full stimulation of the ETC. After that, we added rotenone, an inhibitor of complex I. (0.5 μM). Then, duroquinol (500 μM, Tokyo Chemical Industry, Tokyo, Japan) was added to investigate complex III, followed by the addition of the complex III inhibitor antimycin A (2.5 μM). In a second run, complexes II and IV were analyzed using succinate (10 mM) as substrate in the presence of rotenone (0.5 μM) to block complex I. This was followed by the addition of ADP (2 mM). Then, we added oligomycin (2.5 μM) for the determination of the leak-state respiration. This was followed by the addition of FCCP (1 μM) to reach a full stimulation of the ETC. Then, we added antimycin A (2.5 μM) in order to block the electron transport between complex II and IV and finally N, N, N′, N′ tetramethyl-p -phenylenediamine dihydrochloride (TMPD) and ascorbate (0.5 and 2 mM, respectively) as artificial substrates of complex IV before the addition of the complex IV inhibitor KCN (1 mM).

To assure the integrity of the outer mitochondrial membrane, the absence of a stimulatory effect of exogenous cytochrome c (10 μM) on respiration was demonstrated before the addition of KCN. We used the Pierce bicinchoninic acid (BCA) protein assay kit from Merck (Darmstadt, Germany) to determine the protein concentrations. Respiration rates are expressed in picomoles O_2_ per second per mg protein.

### Mitochondrial superoxide anion accumulation

Mitochondrial superoxide anion production was assessed with the fluorogenic, mitochondria-specific dye MitoSOX^™^ Red reagent (Molecular Probes, Eugene, OR, USA). Isolated mitochondria (200 μg) were stained with MitoSOX red (dissolved in DPBS) at a final concentration of 2 μM. After addition of MitoSox, the plate was incubated in the dark at 37°C for 15 min, afterwards fluorescence was measured at an excitation wavelength of 510 nm and an emission wavelength of 580 nm using a Tecan Infinite pro 200 microplate reader. We normalized the results to the protein content using the Pierce BCA Protein Assay Kit according to manufacturer’s instruction.

### Tissue GSH content

The reduced GSH content was determined using the luminescent GSH-Glo Glutathione assay (Promega, Wallisellen, Switzerland) according to the manufacture’s manual. Liver tissue was homogenized with a Micro-dismembrator during 1 min at 2,000 rpm (Sartorius Stedim Biotech, Göttingen, Germany). 100 μL GSH-Glo Reagent was added to 50 mg liver and incubated for 30 min in the dark. Afterwards, 100 μl Luciferin Detection Reagent was added to each 96-well and the luminescence was measured after 15 min in the dark using a Tecan M200 Pro Infinity plate reader (Männedorf, Switzerland).

### Measurement of thiobarbituric acid-reactive substances

The measurement of plasma thiobarbituric acid-reactive substances (TBARS) was based on the formation of malondialdehyde using a commercially available TBARS Assay Kit (Cayman Chemical, USA), according to the manufacturer’s instructions.

### Mitochondrial DNA content

To measure the mitochondrial DNA content, we determined the ratio of one mitochondrial gene to a nuclear gene using quantitative real-time RT-PCR as described previously with some modifications [26]. Total DNA was extracted using the DNeasy Blood and Tissue Kit (Qiagen) following the manufacturer’s instructions (Quick-Start Protocol). The concentration of the extracted DNA was measured spectrophotometrically at 260 nm with the NanoDrop 2000 (Thermo Scientific, Wohlen, Switzerland). Afterwards, DNA was diluted in RNase free water to a final concentration of 10 ng/μL. The analysis of the mitochondrial ND1 and the nuclear 36B4 genes was performed using SYBR Green real-time PCR (Roche Diagnostics, Rotkreuz, Switzerland) on an ABI PRISM 7700 sequence detector (PE Biosystems, Rotkreuz, Switzerland). Quantification was performed using the comparative-threshold cycle method. The list of primers purchased from Microsynth (Balgach, Switzerland) and used in this study can be found in S1 Table.

### Statistical methods

Data are given as the mean ± S.E.M. Statistical analyses were performed using the Student t-test using GraphPad Prism 6 (GraphPad Software, La Jolla, CA, US). P-values <0.05 (*), <0.01 (**) or <0.001 (***) were considered as significant.

## Results

### Dose finding

**Fig 1:**
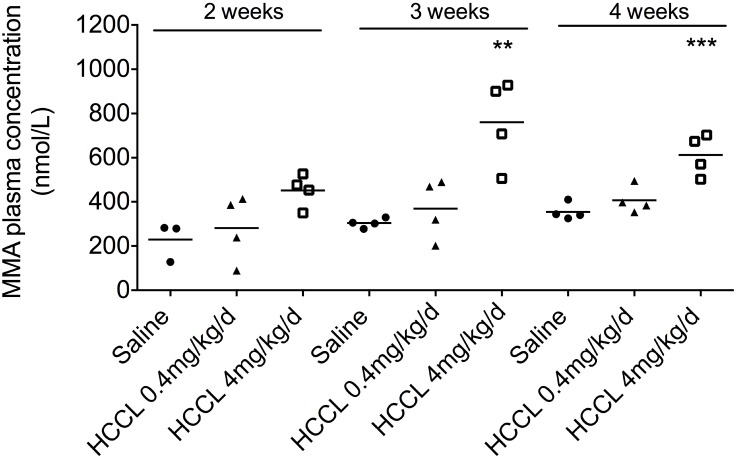
MMA plasma concentration. MMA levels were measured with LC-MS/MS analysis using the ClinMass^®^ Complete Kit after mice were treated with HCCL at 0.4 mg/kg/d or 4 mg/kg/d for two, three or four weeks. Data are expressed as the mean and individual values. **p < 0.01, ***p < 0.001.

The goal of this first part of the study was to determine the optimal dose and duration of HCCL treatment by determination of the MMA plasma concentration. As shown in Fig 1, treatment with 0.4 mg/kg HCCL per day showed only a non-significant increase of plasma MMA concentrations. In comparison, treatment with 4 mg/kg HCCL per day increased the plasma MMA concentration significantly with the increase being maximal (153% increase compared to control mice) already at 3 weeks. We therefore decided to perform the following animal study with HCCL at 4 mg/kg/d for 3 weeks.

### Characterization of the animals

**Fig 2:**
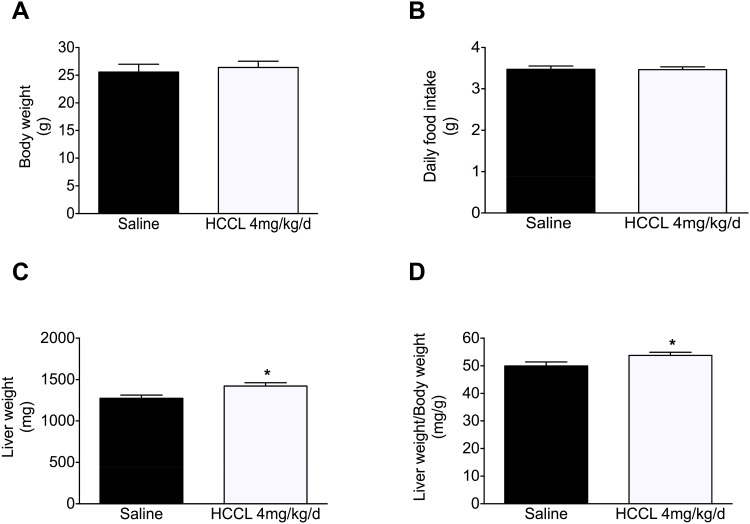
Characterization of the animals. (A) Body weight. (B) Daily food intake. (C) Liver weight. (D) Liver weight/body weight ratio. Data are expressed as mean ± SEM. *p < 0.05.

Body weight (Fig 2A) and daily food intake (Fig 2B) were similar in both groups. The absolute liver weight was 11% increased in the HCCL-treated compared to saline-treated control animals (Fig 1C; p<0.05). This increase in liver weight was also detectable when the liver weight was expressed relative to the body weight (Fig 2D; p<0.05). We found that the MMA plasma concentration was higher in the HCCL-treated compared to control mice (757±60 vs 493±24 nmol/L in HCCL-treated vs. control mice, respectively; p<0.01).

Plasma AST activity and total bilirubin concentrations were slightly higher in the HCCL treated mice compared to the saline treatment, but without reaching statistical significance (Table 1).

**Table 1:** Plasma parameters.

	Saline	HCCL 4mg/kg/d
**AST (U/L)**	19±1	22±2
**Total Bilirubin (g/L)**	0.7±0.1	1.1±0.2

Aspartate aminotransferase (AST) activity and total bilirubin concentration were determined in plasma of mice treated with saline or HCCL 4mg/kg i.p. for 3 weeks.

Data are expressed as mean ± SEM.

### Liver histology and hepatocyte proliferation

In agreement with serum bilirubin and transaminases, which were not significantly different between the groups, there were no gross pathological changes in the livers from HCCL-treated animals by hematoxylin eosin staining (results not shown).

**Fig 3:**
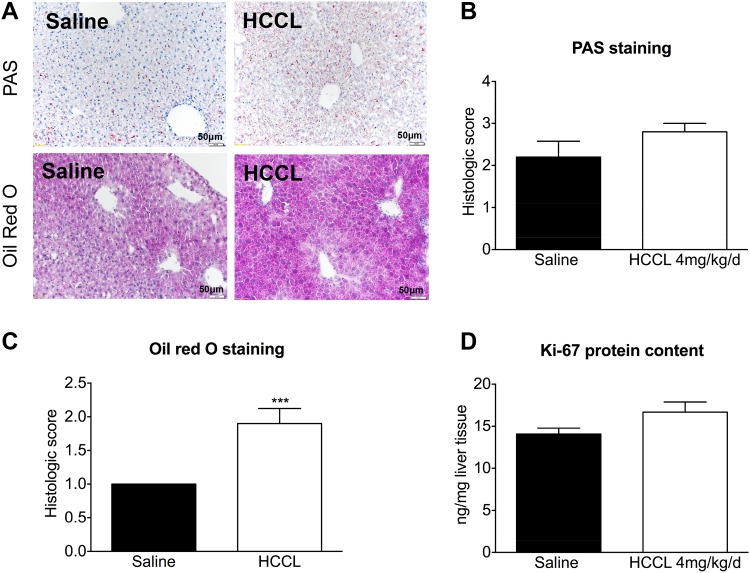
Cell proliferation and histological stainings. (A) PAS and oil red stainings of liver sections from HCCL-treated (4 mg/kg/day) and control mice. (B) Histological quantification of PAS staining (reflecting liver glycogen) (C) Histological quantification of oil red staining (reflecting lipids). (D) Ki-67 protein content as a marker for cell proliferation. Data are expressed as mean ± SEM. ***p < 0.001.

The glycogen content in the cytoplasm of hepatocytes was colored and quantified by periodic acid-Schiff (PAS) staining. PAS staining suggested a 25% increase in hepatic glycogen in HCCL-treated compared to control mice, but this increase did not reach statistical significance (Fig 3A and 3B).

Neutral hepatic lipids, mainly triglycerides, were detected and quantified in frozen liver sections using oil red O staining. Similar to the results of hepatic glycogen, the triacylglycerol content was higher in HCCL-treated compared to control mice (90% increase compared to control mice), reaching statistical significance (Fig 3A and 3C).

Beside storage of glycogen and/or fat, cell proliferation could also have been a cause for the observed increase in liver weight in HCCL-treated mice. We therefore determined the hepatic protein content of Ki-67 as marker of hepatocyte proliferation [27]. After treatment with HCCL, the Ki-67 protein expression was 19% higher in livers of treated as compared to control mice. This increase did not achieve statistical significance (p = 0.08; Fig 3D).

### Acute effect of methylmalonyl-CoA on CPT1 activity in liver mitochondria

**Fig 4:**
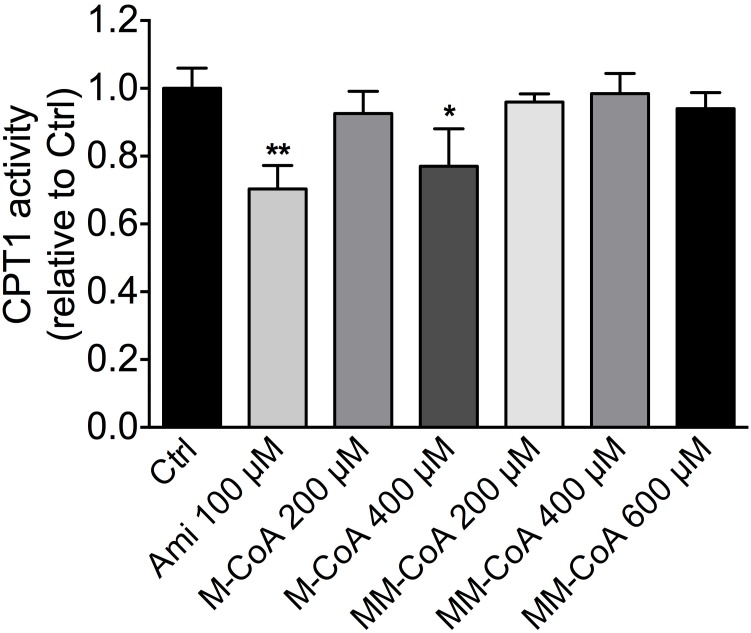
CPT1 activity. Mouse liver mitochondria from control mice were acutely exposed to increased concentrations of MM-CoA. Amiodarone and malonyl-CoA (M-CoA) were used as positive controls. Values represent CPT1 activity relative to the control of at least three independent experiments. Differences between the control and incubations containing toxicants were calculated with a one-way ANOVA followed by a Dunnett’s post test. Statistical significance is indicated as *p < 0.05 or **p < 0.01.

In order to find out possible reasons for lipid accumulation in hepatocytes, we assessed the activity of carnitine palmitoyltransferase 1 (CPT1), the rate limiting enzyme of hepatic fatty acid beta-oxidation, in isolated liver mitochondria exposed to MM-CoA. As expected, the positive controls 100 μM amiodarone and 400 μM malonyl-CoA decreased CPT1 activity significantly (p<0.01 and p<0.05 respectively; Fig 4). However, the CPT1 activity was not affected by increasing concentrations of methylmalonyl-CoA. These results showed that the observed lipid accumulation was not due to the inhibition of CPT1 by MM-CoA.

### Mitochondrial membrane potential (Δψ_m_) and activity of the electron transport chain

**Fig 5:**
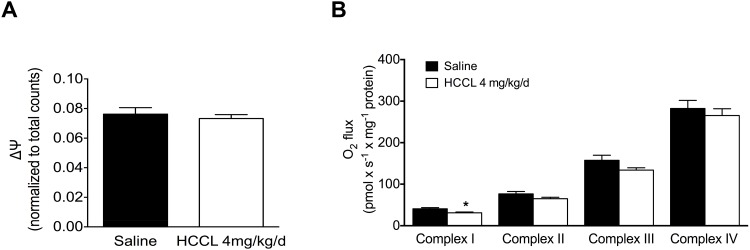
Mitochondrial function. (A) Mitochondrial membrane potential (Δψ_m_) was measured with the [phenyl-^3^H]-tetra-phenylphosphonium bromide uptake assay in isolated liver mitochondria of HCCL-treated and control mice. (B) Electron transport chain complex activities in mouse liver homogenate of both groups. After addition of substrates and inhibitors of the different complexes, complex activities were determined on an Oxygraph-2k high- resolution respirometer. HCCL: hydroxy-cobalamin[c-lactam]. Data are expressed as mean ± SEM. *p < 0.05.

Since HCCL is an established mitochondrial toxicant in rats [14], we next assessed the impact of HCCL treatment on oxidative metabolic function of HepG2 cells. The mitochondrial membrane potential (Δψ_m_) is an important parameter of mitochondrial integrity, since the electrical potential across the inner mitochondrial membrane is built up mainly by the proton gradient generated by the activity of the complexes of the electron transport chain [28]. HCCL treatment decreased the mitochondrial membrane potential only numerically (by 5%), but statistically significant (Fig 5A).

We next assessed the activity of complexes I-IV of the electron transport chain by measuring the oxygen consumption in the presence of different substrates (Fig 5B). The treatment with HCCL significantly reduced the activity of complex I by 31% (p<0.05). The activity of complex II (11% reduction), III (15% reduction) and IV (7% reduction) were also numerically reduced but without reaching statistical significance.

### Oxidative stress and mitochondrial proliferation

**Fig 6:**
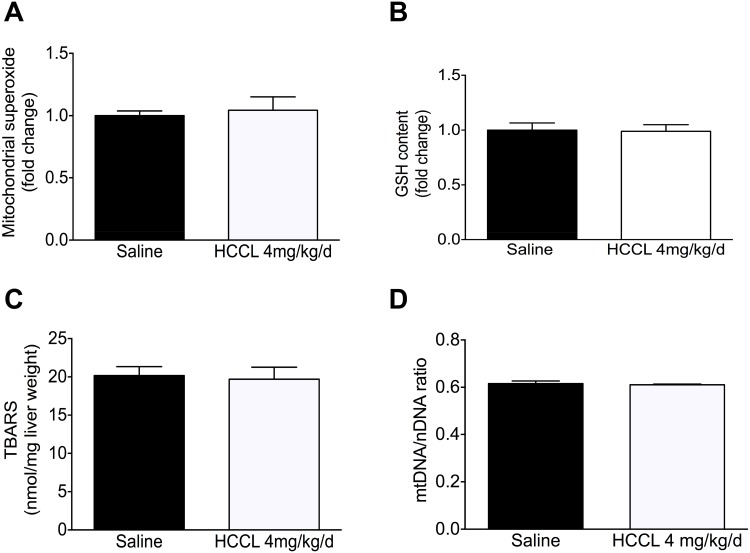
Evaluation on oxidative stress. (A) Mitochondrial superoxide production was measured with MitoSOX^™^ Red reagent in isolated liver mitochondria in mice treated or not with HCCL. (B) Reduced glutathione (GSH) in the liver of HCCL-treated and control mice, determined with the luminescent GSH-Glo Glutathione assay. (C) Hepatic concentrations of TBARS of mice based on the formation of malondialdehyde. (D) DNA expression of a mitochondrial (ND-1) and a nuclear gene (36B4) determined by qRT-PCR. Data are expressed as mean ± SEM.

It is well established that damage of the electron flow across the mitochondrial electron transport chain is associated with cellular ROS accumulation [29]. However, despite the observed damage to complex I activity, superoxide anion accumulation was only 5% higher in liver mitochondria of HCCL-treated compared to controls, not reaching statistical significance (Fig 6A).

In agreement with this finding, we also did not observe a significant drop in the hepatic glutathione pool (Fig 6B) or in the accumulation hepatic of lipid peroxides (as reflected by the hepatic accumulation of thiobarbituric acid reactive substances, TBARS) (Fig 6C).

Impaired function of complexes of the respiratory chain cannot only be associated with ROS accumulation, but also with mitochondrial proliferation [15, 30, 31]. The ratio of the mtDNA to the nuclear DNA provides a measure of mitochondrial DNA homeostasis and represents a marker of mitochondrial proliferation [32]. As shown in Fig 6D, qRT-PCR analysis did not show significant alterations in the ratio of mtDNA/nDNA after HCCL treatment.

## Discussion

In the current study, we showed that HCCL treatment for 3 weeks at 4 mg/kg/d i.p. in mice 1) caused an elevated plasma concentration of MMA, 2) significantly increased the liver weight, which was correlated primarily with accumulation of lipids and less so with glycogen as well as cell proliferation, and 3) altered mitochondrial function as evidenced by impaired activity of complex I of the respiratory chain.

We sought to translate the established rat HCCL model [14, 15, 33] into mice, since we assumed that the amount of HCCL used could be reduced and mice are more practical to use than rats for toxicological studies. A mouse model of impaired hepatic mitochondrial function could be useful to detect and characterize mitochondrial toxicants [34, 35]. In a first step, we performed a dose-finding study in which we treated mice with HCCL at two different doses (0.4 mg/kg and 4 mg/kg subcutaneously per day) for three different durations (two, three and four weeks). We could show that the plasma concentration of MMA, a marker of MM-CoA mutase activity, reached the highest level with a daily HCCL dose of 4 mg/kg after 3 weeks of treatment. According to these results, we decided to perform a second study in mice using this dosage and treatment duration in order to characterize these mice metabolically.

A dose of 4 mg/kg corresponds roughly to 80 μg per mouse per day, which is higher than the doses used in rats (48 μg per rat per day in the studies by Krahenbuhl et al. [14, 15] or approximately 0.5 μg per day in the study by Saido et al. [36]. The higher dose necessary to reach a similar metabolic response in mice compared to rats may be due to a lower sensitivity of mice to HCCL than rats or due to the daily intraperitoneal administration of HCCL, which may lead to higher renal losses and may have different effects on mitochondria than a continuous infusion via osmotic minipumps.

In the second study, we observed that HCCL 4 mg/kg for 3 weeks significantly increased the liver weight. In order to explain this finding, we investigated the accumulation of the major metabolites such as glycogen and fat (triglycerides) as well as hepatocyte proliferation. We found a 90% increase in the hepatic fat content in HCCL-treated mice, whereas the glycogen content and hepatocyte proliferation were not significantly increased. The hepatic accumulation of triglycerides is explained best by the impaired activity of complex I of the electron transport chain. During breakdown of fatty acids, NADH is generated, which is a substrate of complex I of the electron transport chain. Impaired complex I activity is therefore associated with reduced fatty acid metabolism [37]. We excluded the possibility that hepatic accumulation of MM-CoA may inhibit CPT1, the rate-limiting enzyme of hepatic fatty acid metabolism, by showing that CPT1 activity in isolated mitochondria is not impaired by MM-CoA.

Our studies showed that treatment with HCCL at 4 mg/kg for 3 weeks was associated with a significant impairment of the activity of complex I of the mitochondrial electron transport chain and a numerical decrease of the activity of complex II and III. In comparison, long-term treatment with HCCL in rats was associated with impaired activities of complex III and IV in isolated liver mitochondria [14]. These divergences may be explained by species differences and/or by differences in the application method. As explained above, the intermittently high plasma concentrations associated with the daily intraperitoneal application of HCCL in the current study may have influenced the mitochondrial metabolism differently compared to the continuous administration with osmotic minipumps.

Since impaired activity of the electron transport chain is associated with increased mitochondrial production of ROS [29], we expected mitochondrial accumulation of ROS. However, we were surprised to find no significant changes in mitochondrial superoxide accumulation. In contrast to the current findings, we have shown recently that treatment of HepG2 cells with HCCL is associated with increased mitochondrial ROS production [38]. One explanation for these divergences could be the absence of specific mitochondrial substrates (i.e. glutamate/malate or succinate) in the current study; addition of substrates would have increased mitochondrial oxygen consumption and ROS production. We also quantified indirect markers of oxidative stress such as reduced glutathione (GSH) and lipid peroxidation. In agreement unaltered ROS accumulation, the hepatic GSH pool was maintained and lipid peroxidation was not increased. Furthermore, also the mitochondrial DNA content was not affected by HCCL, excluding mitochondrial proliferation as a compensatory reaction to impaired activity of the electron transport chain. This differs from rats treated with HCCL, where proliferation of hepatic mitochondria was described [15]. The results of the current study indicate that the antioxidative capacity in the hepatocytes of HCCL-treated mice was large enough to trap the increased mitochondrial ROS production associated with impaired complex I activity.

An aim of the study was to create a simple, reproducible animal model of hepatic mitochondrial dysfunction usable for toxicological studies. Since adverse hepatic drug reactions often affect mitochondrial function [39], preexisting mitochondrial dysfunction could represent a susceptibility factor leading to the manifestation or aggravation of liver toxicity. This has been demonstrated for instance for valproate-associated liver failure, where mutations in the gene coding for DNA polymerase gamma are an established susceptibility factor [19]. Since treatment of mice with HCCL is associated with a decreased activity of complex I of the electron transport chain in hepatocytes, mice treated with HCCL could represent a model for studying mitochondrial toxicants. However, HCCL-associated impairment of complex I inhibition was not strong enough to result in mitochondrial ROS accumulation and lipid peroxidation for which reason it is questionable whether HCCL-treated mice are a suitable model for hepatic mitochondrial dysfunction. Further studies are necessary to answer this question.

In conclusion, mice exposed to intraperitoneal HCCL at 4 mg/kg day for 3 weeks showed elevated MMA plasma concentrations, an increase in liver weight mainly due to triglyceride accumulation and an impaired activity of complex I of the electron transport chain of liver mitochondria. Further studies have to address the question whether this model is suitable for studying liver toxicity of mitochondrial toxicants.

## Supporting Information

S1 Table Primer sequences. Primer sequences used for quantitative Real-Time PCR amplification.
(DOCX)

## Abbreviations

Δψ_m_: Mitochondrial membrane potential
CPT1: Carnitine palmitoyltransferase 1
DMSO: Dimethyl sulfoxide
GSH: Reduced glutathione
H_2_O_2_: Hydrogen peroxide
HCCL: Hydroxy-cobalamin[c-lactam]
HPLC: High-performance liquid chromatography
M-CoA: Malonyl-CoA
MMA: Methylmalonic acid
MM-CoA mutase: Methylmalonyl-CoA mutase
ROS: Reactive oxygen species
SOD: Superoxide dismutase
TBARS: Thiobarbituric acid-reactive substances
TMPD: N, N, N’, N’-tetramethyl-p-phenylenediamine
