# Supplementary material for: Hepatic Effects of Pharmacological Doses of Hydroxy-Cobalamin[c-lactam] in Mice
Source: PLoS One. 2017 Jan 30;12(1):e0171026. doi: 10.1371/journal.pone.0171026 (PMC5279765; doi:10.1371/journal.pone.0171026)
Supplement: S1 Table — Primer sequences used for quantitative Real-Time PCR amplification. (DOCX) [file pone.0171026.s001.docx]

| **Target gene** | **Sequence 5’--🡪3’** |
| --- | --- |
| ND-1 | ATG GCC AAC CTC CTA CTC CT  CTA CAA CGT TGG GGC CTT T |
| 36B4 | GGA ATG TGG GCT TTG TGT TC  CCC AAT TGT CCC CTT ACC TT |

**S1 Table**
